# Evaluating a guideline-aligned prednisone threshold within the Lupus Low Disease Activity State (LLDAS): associations with damage accrual

**DOI:** 10.1136/rmdopen-2026-007162

**Published:** 2026-09-23

**Authors:** Ioannis Parodis, Rama Andraos, Matteo Bottai, Christopher Sjöwall

**Affiliations:** 1 Division of Rheumatology, Department of Medicine Solna, Karolinska Institutet, Karolinska University Hospital, and Center for Molecular Medicine (CMM), Stockholm, Sweden; 2 Department of Rheumatology, Faculty of Medicine and Health, Örebro University, Örebro, Sweden; 3 Division of Inflammation and Infection/Rheumatology, Department of Biomedical and Clinical Sciences, Linköping University, Linköping, Sweden; 4 Division of Biostatistics, Department of Environmental Medicine, Karolinska Institutet, Stockholm, Sweden; 5 Department of Biomedical and Clinical Sciences, Division of Inflammation and Infection/Rheumatology, Linköping University, Linköping, Sweden

**Keywords:** Systemic Lupus Erythematosus, Lupus Erythematosus, Systemic, Outcome Assessment, Health Care, Outcome and Process Assessment, Health Care, Autoimmunity

## Abstract

**Objective:**

To examine whether a modified Lupus Low Disease Activity State with a prednisone-equivalent threshold ≤5 mg/day (LLDAS-5) is more strongly associated with lower damage accrual than conventional LLDAS using a ≤7.5 mg/day threshold (LLDAS-7.5) in systemic lupus erythematosus (SLE).

**Methods:**

We analysed 332 patients, including 105 incident cases. For each patient, yearly Systemic Lupus International Collaborating Clinics/American College of Rheumatology Damage Index (SDI) worsening occasions and average proportion of follow-up spent in LLDAS-5 and LLDAS-7.5 were derived. Patient-level Poisson regression with follow-up years as the exposure and Huber’s robust variance estimator estimated rate ratios (RRs). Parallel models were fitted for the two definitions. Full-cohort multivariable models adjusted for age, sex, ethnicity, SLE duration, baseline Systemic Lupus Erythematosus Disease Activity Index 2000 and baseline SDI.

**Results:**

In the full cohort, 142/332 patients experienced at least one SDI increase (190 occasions). Crude RRs were 0.61 (95% CI 0.37 to 1.00; p=0.052) for LLDAS-5 and 0.73 (95% CI 0.39 to 1.37; p=0.332) for LLDAS-7.5. Multivariable RRs were 0.56 (95% CI 0.37 to 0.86; p=0.007) and 0.47 (95% CI 0.27 to 0.85; p=0.012), respectively, with similar model fit. Among incident cases, 29/105 patients accrued damage (29 occasions); both LLDAS-5 (RR 0.31, 95% CI 0.11 to 0.89; p=0.029) and LLDAS-7.5 (RR 0.29, 95% CI 0.09 to 0.93; p=0.036) were associated with lower damage accrual.

**Conclusion:**

Longer time in LLDAS-5 was robustly associated with less damage accrual. Crude full-cohort analyses suggested a numerically stronger inverse association and slightly better model fit for LLDAS-5, but this apparent advantage was not sustained after multivariable adjustment. These findings support LLDAS-5 as a clinically meaningful, guideline-aligned target, while its incremental prognostic value over conventional LLDAS-7.5 requires further study.

**WHAT IS ALREADY KNOWN ON THIS TOPIC**

- Lupus Low Disease Activity State (LLDAS) is an established treatment target in systemic lupus erythematosus and the original definition allows prednisone doses up to 7.5 mg/day.
- More recent management recommendations advocate tapering maintenance glucocorticoids to ≤5 mg/day and complete withdrawal whenever possible.

**WHAT THIS STUDY ADDS**

- Longer time in both LLDAS with a prednisone-equivalent threshold ≤5 mg/day (LLDAS-5) and conventional LLDAS using a ≤7.5 mg/day threshold (LLDAS-7.5) was associated with less damage accrual.
- In crude full-cohort analyses, LLDAS-5 showed a numerically stronger inverse association and slightly better model fit, but this apparent advantage was not sustained after multivariable adjustment.

**HOW THIS STUDY MIGHT AFFECT RESEARCH, PRACTICE OR POLICY**

- The findings support LLDAS-5 as a clinically meaningful, guideline-aligned target and reinforce the priority of glucocorticoid minimisation.
- The crude signal favouring LLDAS-5 warrants further study, while the present data do not establish incremental prognostic superiority over conventional LLDAS-7.5.

## Introduction

 Lupus Low Disease Activity State (LLDAS) is a validated, clinically and biologically relevant therapeutic target in systemic lupus erythematosus (SLE) and the original definition includes a prednisone-equivalent threshold of ≤7.5 mg/day (LLDAS-7.5).^1–3^ More recent SLE management recommendations have shifted towards more stringent restriction of long-term glucocorticoid exposure, with a goal of ≤5 mg/day and complete withdrawal when feasible.^4 5^

Since chronic glucocorticoid exposure contributes to irreversible organ damage,^6 7^ it is clinically relevant to ask whether a more stringent LLDAS definition using a guideline-aligned prednisone-equivalent threshold of ≤5 mg/day (LLDAS-5)^8–10^ identifies a state that is more strongly associated with lower damage accrual. We therefore compared the association between time spent in LLDAS-5 and in conventional LLDAS-7.5 and worsening in the Systemic Lupus International Collaborating Clinics (SLICC)/American College of Rheumatology (ACR) Damage Index (SDI).^11^

## Methods

The *Clinical Lupus Register in North-eastern Gothia* (Swedish acronym: ‘KLURING’) was established in early 2008 and includes all adult patients (≥18 years of age) with a confirmed diagnosis of SLE, meeting the 1982 ACR and/or the 2012 SLICC classification criteria,^12 13^ in Region Östergötland, Sweden.^14 15^ KLURING contains both prevalent cases, who were diagnosed before registry enrolment, and incident cases, who were enrolled at the time of SLE diagnosis. Thus, SLE duration at inclusion represents the interval between diagnosis and registry enrolment and is, by definition, zero for incident cases. At the time of data extraction for the present study, KLURING comprised 332 unique patients with SLE (2926 visits), 105 of whom enrolled at the time of SLE diagnosis (incident cases; 745 visits) and longitudinal data were available from enrolment until data extraction (October 2021).^16^

For each patient, all yearly occasions on which the SDI increased were counted and summed over follow-up to create one patient-level count outcome. Follow-up did not stop after the first SDI increase. Yearly average proportions of follow-up spent in LLDAS-5 and in conventional LLDAS-7.5 were derived. Each patient contributed one observation to the Poisson regression, with the number of observed follow-up years entered as the exposure. Huber’s robust variance estimator was applied to the regression parameters. Analyses were performed in the full cohort and in incident cases only.

Since LLDAS-5 is nested within conventional LLDAS-7.5, the primary comparison used separate, parallel Poisson models rather than entering the two nested exposure measures simultaneously. Crude models were fitted for each definition in the full cohort and incident cases. In the full cohort, multivariable models additionally adjusted for age at inclusion, sex, white/Caucasian ethnicity, SLE duration at inclusion, baseline Systemic Lupus Erythematosus Disease Activity Index 2000 (SLEDAI-2K) and baseline SDI. Model fit was compared using Akaike’s information criterion (AIC) and Bayesian information criterion (BIC). Given the limited number of damage events among incident cases, a fully adjusted model was not fitted in this subgroup. As a secondary analysis addressing the glucocorticoid-dose component of LLDAS, conventional LLDAS-7.5 was decomposed into mutually exclusive time in LLDAS-5 and time meeting LLDAS while receiving >5 to ≤7.5 mg/day prednisone equivalent (LLDAS >5). Correlations and variance inflation factors (VIFs) were calculated to assess collinearity. Data are presented as RR, 95% CI and p value, with p*<*0.05 denoting statistical significance. To explore whether the glucocorticoid-dose components differed in underlying disease activity, an additional exploratory analysis modelled SLEDAI-2K in relation to the mutually exclusive LLDAS-5 and LLDAS >5 components, with robust SEs clustered by patient.

### Patient and public involvement

Patient research partners (Yvonne Enman and Karin Blomkvist Sporre) were involved in the design and dissemination plans of the study. The public was not involved in the design, or conduct, or reporting, or dissemination plans of this research.

## Results

### Patient characteristics

Clinical and laboratory characteristics of the full study population and the subgroup of incident cases at index date are summarised in table 1. During follow-up, 142 of 332 patients (42.8%) in the full cohort experienced at least one SDI increase, with 190 yearly SDI-worsening occasions in total. Among the 105 incident cases, 29 patients (27.6%) accrued damage, with 29 yearly worsening occasions.

**Table 1:** Characteristics of study population

Variables	Full cohort N=332	Incident cases N=105
Women, n (%)	284 (85.5)	85 (80.9)
White/Caucasian, n (%)	293 (88.3)	88 (83.8)
Age at SLE diagnosis, years, mean (SD)	40.0 (17.5)	44.3 (17.0)
Age at inclusion, years, mean (SD)	48.7 (17.6)	44.3 (17.0)
SLE duration at inclusion, years, median (IQR)	4.1 (12.8)	0 (0.0)
Tobacco smoking at or until inclusion, n (%)	146 (44.0)	31 (29.5)
APS diagnosed until data extraction, n (%)	55 (16.6)	14 (13.3)
Total follow-up time, years, mean (SD)	6.9 (4.2)	4.9 (3.5)
Percentage of time spent in LLDAS-5, mean (SD)	74.3 (31.5)	66.1 (35.6)
Percentage of time spent in LLDAS-7.5, mean (SD)	78.7 (28.6)	69.6 (33.5)
**Baseline characterisation and treatment**		
SLEDAI-2K score, median (IQR)	2 (2–6)	6 (2–9)
Clinical SLEDAI-2K score, median (IQR)	0 (0–4)	3 (0–6)
Meeting LLDAS-7.5 definition, n (%)	221 (66.6)	39 (37.1)
Meeting LLDAS-5 definition, n (%)	206 (62.0)	38 (36.2)
PhGA, scale 0–3, median (IQR)	0 (0–1)	1 (0–2)
SDI score, median (IQR)	0 (0–1)	0 (0–0)
SDI score >0, n (%)	137 (41.3)	0 (0.0)
Glucocorticoid use, n (%)	222 (66.9)	70 (66.6)
Prednisone-equivalent dose, mg/day, median (IQR)	5.0 (0.0–7.5)	5 (2.5–20.0)
Antimalarial therapy, n (%)	241 (72.6)	97 (92.3)
Conventional immunosuppressants, n (%)	169 (50.9)	41 (39.0)
Biological therapy, n (%)	17 (5.1)	5 (4.8)
**Baseline laboratory data**		
C3, g/L, mean (SD)	0.97 (0.28)	0.93 (0.14)
C4, g/L, mean (SD)	0.18 (0.09)	0.15 (0.08)
Anti-dsDNA (+), n (%)	169 (50.9)	63 (60.0)
Anti-Sm (+), n (%)	49 (14.8)	16 (15.2)
**SLE classification** (1982 ACR criteria; cumulative)		
Number of fulfilled criteria, mean (SD)	4.8 (1.3)	4.7 (1.2)
Malar rash, n (%)	125 (37.7)	30 (28.6)
Discoid lupus, n (%)	51 (15.4)	14 (13.3)
Photosensitivity, n (%)	173 (52.1)	53 (50.5)
Oral ulcers, n (%)	38 (11.4)	11 (10.5)
Arthritis, n (%)	254 (76.5)	75 (71.4)
Serositis, n (%)	118 (35.5)	28 (26.7)
Renal disorder, n (%)	92 (27.7)	31 (29.5)
Neurological disorder, n (%)	20 (6.0)	7 (6.7)
Haematological disorder, n (%)	204 (61.4)	68 (64.8)
Immunological disorder, n (%)	186 (56.0)	72 (68.6)
Antinuclear antibody (+), n (%)	328 (98.8)	104 (99.0)

Data are presented as n (%), mean (SD) or median (IQR).

(+), positive levels; ACR, American College of Rheumatology; Anti-dsDNA, anti-double-stranded DNA; Anti-Sm, anti-Smith; APS, antiphospholipid syndrome; C3, complement protein 3; C4, complement protein 4; LLDAS-5, Lupus Low Disease Activity State using a prednisone-equivalent threshold of ≤5 mg/day; LLDAS-7.5, conventional Lupus Low Disease Activity State using a prednisone-equivalent threshold of ≤7.5 mg/day; PhGA, Physician’s Global Assessment; SDI, Systemic Lupus International Collaborating Clinics/American College of Rheumatology Damage Index; SLE, systemic lupus erythematosus; SLEDAI-2K, Systemic Lupus Erythematosus Disease Activity Index 2000.

### Association between time in LLDAS and damage accrual

In the full cohort, longer time in LLDAS-5 was associated with a lower yearly rate of SDI worsening in the crude model (RR 0.61; 95% CI 0.37 to 1.00; p=0.052), while the corresponding estimate for conventional LLDAS-7.5 was RR 0.73 (95% CI 0.39 to 1.37; p=0.332) (table 2; figure 1). The crude LLDAS-5 model had slightly lower AIC/BIC than the conventional LLDAS-7.5 model (642.5/650.1 vs 645.1/652.7). After multivariable adjustment, both definitions were significantly associated with lower damage accrual: RR 0.56 (95% CI 0.37 to 0.86; p=0.007) for LLDAS-5 and RR 0.47 (95% CI 0.27 to 0.85; p=0.012) for conventional LLDAS-7.5. Model fit was virtually identical (AIC/BIC 575.9/606.3 vs 576.1/606.5, respectively), providing no evidence that lowering the glucocorticoid ceiling improved model performance.

**Figure 1:**
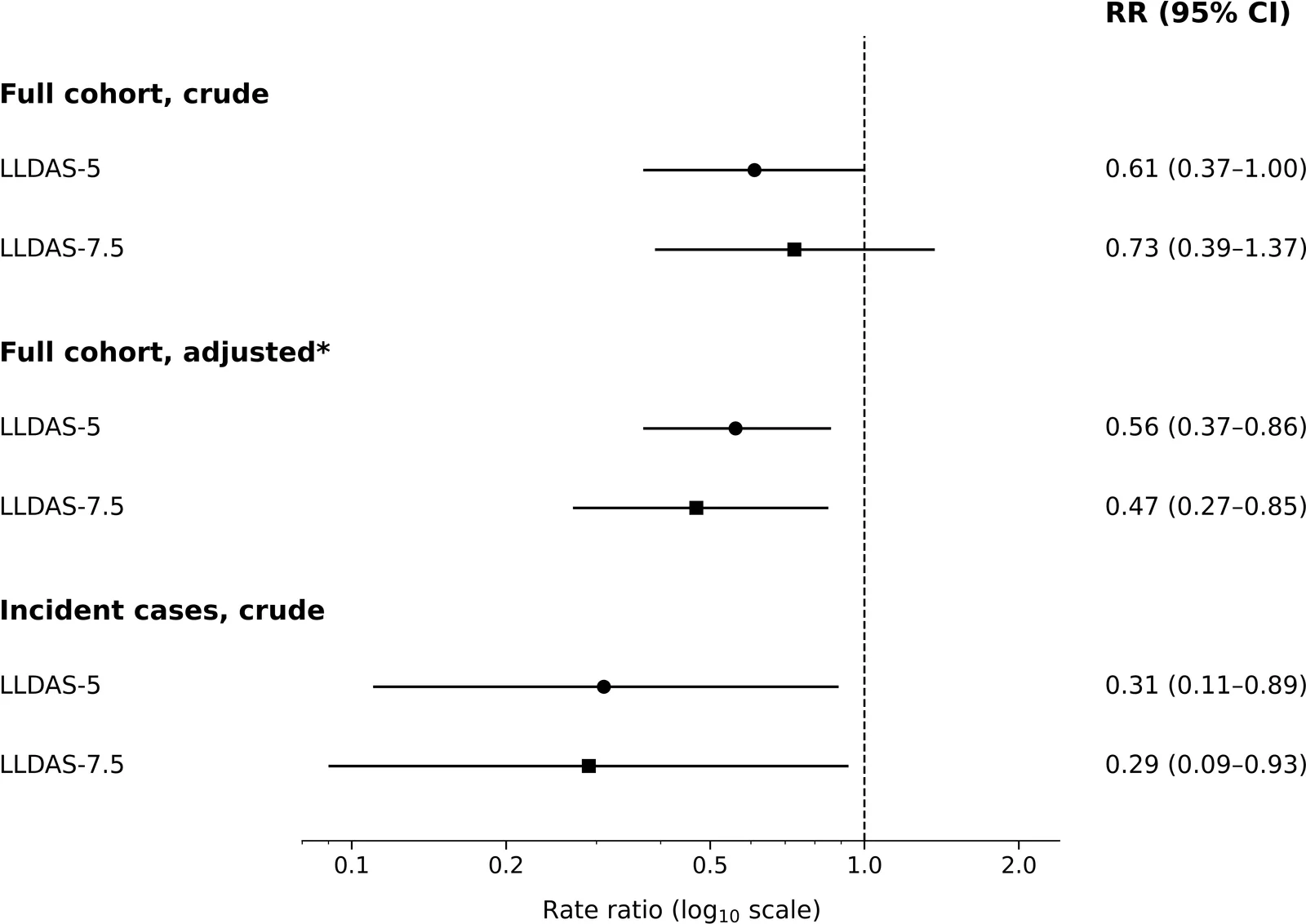
Forest plot showing rate ratios (RRs) and 95% CIs for the association between yearly average time in LLDAS and yearly rate of SDI worsening in parallel models for LLDAS-5 and conventional LLDAS-7.5. *Full-cohort adjusted models include age at inclusion, sex, white/Caucasian ethnicity, SLE duration at inclusion, baseline SLEDAI-2K and baseline SDI. LLDAS-5, Lupus Low Disease Activity State using a prednisone-equivalent threshold of ≤5 mg/day; LLDAS-7.5, conventional Lupus Low Disease Activity State using a prednisone-equivalent threshold of ≤7.5 mg/day; SDI, Systemic Lupus International Collaborating Clinics/American College of Rheumatology Damage Index.

**Table 2:** Association between yearly average time in LLDAS and yearly rate of SDI worsening

Population	Model	Exposure	Rate ratio	95% CI	P value
Full cohort	Crude	Time in LLDAS-5	0.61	0.37 to 1.00	0.052
Full cohort	Crude	Time in LLDAS-7.5	0.73	0.39 to 1.37	0.332
Full cohort	Adjusted*	Time in LLDAS-5	0.56	0.37 to 0.86	0.007
Full cohort	Adjusted*	Time in LLDAS-7.5	0.47	0.27 to 0.85	0.012
Incident cases	Crude	Time in LLDAS-5	0.31	0.11 to 0.89	0.029
Incident cases	Crude	Time in LLDAS-7.5	0.29	0.09 to 0.93	0.036

* Multivariable models adjusted for age at inclusion, sex, white/Caucasian ethnicity, SLE duration at inclusion, baseline SLEDAI-2K and baseline SDI. Fully adjusted models were not fitted in incident cases because only 29 SDI-worsening occasions occurred.

LLDAS-5, Lupus Low Disease Activity State using a prednisone-equivalent threshold of ≤5 mg/day; LLDAS-7.5, conventional Lupus Low Disease Activity State using a prednisone-equivalent threshold of ≤7.5 mg/day; SDI, Systemic Lupus International Collaborating Clinics/American College of Rheumatology Damage Index; SLEDAI-2K, Systemic Lupus Erythematosus Disease Activity Index 2000.

Among incident cases, both definitions showed strong and very similar inverse associations with damage accrual: RR 0.31 (95% CI 0.11 to 0.89; p=0.029) for LLDAS-5 and RR 0.29 (95% CI 0.09 to 0.93; p=0.036) for conventional LLDAS-7.5 (table 2; figure 1). Since only 29 damage-worsening occasions occurred in this subgroup, a fully multivariable incident-case model was not fitted. LLDAS-5 and conventional LLDAS-7.5 were highly correlated in the full cohort (r=0.898; VIF 5.16), supporting the use of parallel rather than mutually adjusted primary models. In the secondary decomposition, LLDAS-5 and LLDAS >5 were less correlated (r=−0.418; VIF 1.21). In a model including both mutually exclusive components, their coefficients differed in the full cohort (p=0.008) but not among incident cases (p=0.428); these exploratory damage estimates were imprecise, particularly in incident cases (online supplemental table S1). In an additional exploratory analysis with SLEDAI-2K as the outcome and patient-clustered robust SEs, the coefficients for LLDAS-5 and LLDAS >5 were similar in the full cohort (−1.57, 95% CI −1.89 to −1.25, p<0.001 and −1.54, 95% CI −2.48 to −0.60, p=0.001, respectively). Among incident cases, the corresponding coefficients were −1.83 (95% CI −2.35 to −1.31; p<0.001) and −0.52 (95% CI −2.03 to 0.98; p=0.494) (online supplemental table S2).

## Discussion

In this longitudinal cohort, longer time in both LLDAS-5 and conventional LLDAS-7.5 was associated with less damage accrual, with the clearest associations among patients followed from SLE diagnosis. In the full cohort, crude analyses suggested a potential advantage for LLDAS-5: its point estimate was more pronounced and model fit was modestly better than for conventional LLDAS-7.5. This apparent advantage was not sustained after multivariable adjustment, in which both definitions were significantly associated with lower damage accrual, conventional LLDAS-7.5 had a numerically lower RR and model fit was essentially equivalent. Among incident cases, the effect estimates were almost identical. Collectively, these findings support the prognostic validity of the guideline-aligned LLDAS-5 definition while stopping short of establishing incremental prognostic superiority over conventional LLDAS-7.5.

These findings are clinically relevant since damage accrual in SLE reflects both disease activity and treatment-related toxicity and is closely linked to mortality, while glucocorticoids are an important contributor to irreversible tissue damage.^6 7 17 18^ A prednisone-equivalent ceiling of ≤5 mg/day is more consistent with current steroid-sparing recommendations than the original 7.5 mg/day cut-off embedded in the original LLDAS.^4 5^ While our results do not argue against glucocorticoid minimisation, they suggest that lowering this single threshold does not necessarily improve the association of the composite LLDAS state with long-term damage.

The high correlation between LLDAS-5 and conventional LLDAS-7.5 (r=0.898; VIF 5.16) underscores the difficulty of mutually adjusting nested definitions. We therefore used parallel primary models and, in a secondary analysis, decomposed conventional LLDAS-7.5 into mutually exclusive LLDAS-5 and LLDAS >5 components. Although the two component coefficients for damage differed in the full cohort, this contrast was not significant among incident cases and CIs were wide. In the exploratory disease-activity analysis, the LLDAS-5 and LLDAS >5 components showed very similar inverse associations with SLEDAI-2K in the full cohort, arguing against a simple explanation in which LLDAS-5 merely identified observations with markedly lower disease activity; however, the LLDAS >5 estimate was imprecise in incident cases. These findings should not be interpreted as evidence that reducing an individual patient’s prednisone-equivalent glucocorticoid dose from >5–7.5 mg/day to ≤5 mg/day itself prevents damage. Patients who remain at >5 to ≤7.5 mg/day may have more severe disease, previous major-organ involvement, poorer treatment response or greater treatment requirements. A recent large multinational analysis from the Asia Pacific Lupus Collaboration similarly found comparable protection against flare, organ damage and mortality with conventional LLDAS and LLDAS-5 and did not support revision of the established glucocorticoid ceiling.^19^

Several limitations must be acknowledged. Although the full-cohort models were adjusted for age, sex, ethnicity, SLE duration, baseline SLEDAI-2K and baseline SDI, residual confounding by time-varying disease activity, major-organ involvement, treatment response and use of immunosuppressive or biologic therapy remains possible. The exposure measures summarise accumulated time in LLDAS over follow-up and may obscure within-patient transitions and the temporal sequence between low disease activity and individual damage events; accordingly, the analyses estimate cumulative longitudinal associations rather than short-term prediction or causal effects. The small number of damage events, particularly the 29 events among incident cases, limited the complexity of subgroup analyses and precluded robust analyses of individual damage domains. Most participants were white/Caucasian, which may limit generalisability. Nonetheless, KLURING comprises well-characterised patients with long-term follow-up, and the study provides a useful real-world comparison of two clinically relevant glucocorticoid thresholds within LLDAS.

Importantly, LLDAS-5 remains conceptually and operationally distinct from remission despite sharing a ≤5 mg/day glucocorticoid ceiling. The 2021 DORIS definition requires clinical SLEDAI-2K=0 and Physician’s Global Assessment <0.5 (on a 0–3 scale), whereas LLDAS permits SLEDAI-2K ≤4 subject to its other restrictions and Physician’s Global Assessment ≤1.^20^ Thus, lowering the glucocorticoid ceiling does not by itself collapse the distinction between low disease activity and remission.

## Conclusion

In this Swedish real-world cohort, longer time in LLDAS-5 was robustly associated with less damage accrual, including after multivariable adjustment in the full cohort; strong associations were also observed among incident cases. Crude full-cohort analyses suggested a numerically stronger inverse association and slightly better model fit for LLDAS-5 than for conventional LLDAS-7.5, but this apparent advantage was not sustained after multivariable adjustment. These findings support LLDAS-5 as a clinically meaningful, guideline-aligned treatment target and reinforce the priority of glucocorticoid minimisation, while larger studies are needed to determine whether LLDAS-5 provides incremental prognostic value over the conventional LLDAS-7.5.

## Supplementary material

10.1136/rmdopen-2026-007162 online supplemental file 1
