# Supplementary material for: Evaluating a guideline-aligned prednisone threshold within the Lupus Low Disease Activity State (LLDAS): associations with damage accrual
Source: RMD Open. 2026 Sep 23;12(3):e007162. doi: 10.1136/rmdopen-2026-007162 (PMC13630167; doi:10.1136/rmdopen-2026-007162)
Supplement: Supplementary file 1 [file rmdopen-12-3-s001.docx]

**SUPPLEMENTARY MATERIAL**

**Supplementary Table S1.** Secondary decomposition of conventional LLDAS-7.5 into mutually exclusive glucocorticoid-dose components.

| **Population** | **Component** | **RR** | **95% CI** | **p value** | **Coefficient contrast p*** | **Collinearity** |
| --- | --- | --- | --- | --- | --- | --- |
| Full cohort | LLDAS-5 | 0.75 | 0.39–1.41 | 0.369 | 0.008 | r=−0.418; VIF=1.21 |
| Full cohort | LLDAS>5 | 1.83 | 0.75–4.45 | 0.185 |  |  |
| Incident cases | LLDAS-5 | 0.31 | 0.09–0.99 | 0.049 | 0.428 |  |
| Incident cases | LLDAS>5 | 0.85 | 0.05–15.19 | 0.913 |  |  |
| Full cohort^†^ | LLDAS-5 | 0.48 | 0.27–0.86 | 0.014 | — |  |
| Full cohort^†^ | LLDAS>5 | 0.69 | 0.30–1.57 | 0.376 | — |  |

*Wald contrast comparing the log-rate-ratio coefficients for LLDAS-5 and LLDAS>5 within the same model. LLDAS>5 denotes time meeting all LLDAS criteria while receiving >5 to ≤7.5 mg/day prednisone equivalent. The two components are mutually exclusive and together comprise conventional LLDAS-7.5. Models include both components simultaneously and use follow-up years as the exposure.

^†^Multivariable full-cohort sensitivity model adjusted for age at inclusion, sex, White/Caucasian ethnicity, SLE duration at inclusion, baseline SLEDAI-2K, and baseline SDI; no formal coefficient contrast was calculated for this adjusted model.

**Supplementary Table S2.** Exploratory association of mutually exclusive LLDAS glucocorticoid-dose components with SLEDAI-2K.

| **Population** | **Component** | **Coefficient** | **95% CI** | **p value** | **Interpretation** |
| --- | --- | --- | --- | --- | --- |
| Full cohort | LLDAS-5 | −1.57 | −1.89 to −1.25 | <0.001 | Lower  SLEDAI-2K |
| Full cohort | LLDAS>5 | −1.54 | −2.48 to −0.60 | 0.001 | Lower  SLEDAI-2K |
| Incident cases | LLDAS-5 | −1.83 | −2.35 to −1.31 | <0.001 | Lower  SLEDAI-2K |
| Incident cases | LLDAS>5 | −0.52 | −2.03 to 0.98 | 0.494 | Imprecise; not significant |

Coefficients are from an exploratory regression analysis with SLEDAI-2K as the outcome and robust standard errors clustered by patient. LLDAS>5 denotes time meeting all LLDAS criteria while receiving >5 to ≤7.5 mg/day prednisone equivalent. The mutually exclusive LLDAS-5 and LLDAS>5 components together comprise conventional LLDAS-7.5. No formal coefficient contrast between the two SLEDAI-2K associations was performed; the analysis is presented to assess whether the components showed materially different associations with disease activity.
